# Fault Diagnosis and Location Method for Stator-Winding Single-Phase Grounding of Large Generator Based on Stepped-Frequency Pulse Injection

**DOI:** 10.3390/s25226875

**Published:** 2025-11-11

**Authors:** Binghui Lei, Shuai Xu, Yang Liu, Weiguo Zu, Mingtao Yu, Yanxun Guo, Lianghui Dong, Zhiping Cheng

**Affiliations:** 1 Baihetan Hydropower Plant, Liangshan 615400, China; lei_binghui@ctg.com.cn (B.L.); liu_yang6@ctg.com.cn (Y.L.); zu_weiguo@ctg.com.cn (W.Z.); yu_mingtao@ctg.com.cn (M.Y.); 2 School of Electrical and Information Engineering, Zhengzhou University, Zhengzhou 450001, China; guoyanxun@zzu.edu.cn (Y.G.); dlh@zzu.edu.cn (L.D.); zpcheng@zzu.edu.cn (Z.C.)

**Keywords:** large hydro-generator, single-phase grounding, fault location, current vector, pule injection

## Abstract

Ensuring the safe operation of large hydro-generators is essential for energy supply and economic development. Stator-winding single-phase grounding faults are among the most common failures in such generators. Conventional protection methods—such as fundamental voltage protection, third-harmonic voltage saturation, and low-frequency injection—lack fault location capability and cannot assess the fault severity. This paper proposes a stepwise variable-frequency pulse injection method for fault diagnosis and location in large hydro-generator stator windings. A finite element model of a salient-pole hydro-generator is established to analyze magnetic flux density and electromotive force distributions under normal and fault conditions, from which fault characteristics are derived. Equivalent circuit models suitable for low- and high-frequency pulse injection are developed. A bidirectional pulse injection circuit and algorithm are designed to identify the fault phase via terminal current vector characteristics, diagnose the faulty branch based on leakage loop equivalent inductance, and locate the fault point using voltage–current signal slopes. Simulation results validate the effectiveness of the proposed diagnostic approach.

## 1. Introduction

Large generators, the principal power source in utility systems, are regarded as critical; their reliability is therefore intensively scrutinized. Insulation failure—responsible for roughly one-third of all generator forced outages—can trigger catastrophic events and heavy losses, so online diagnosis and location of stator-winding insulation faults have become a major research focus.

**Figure 1:**
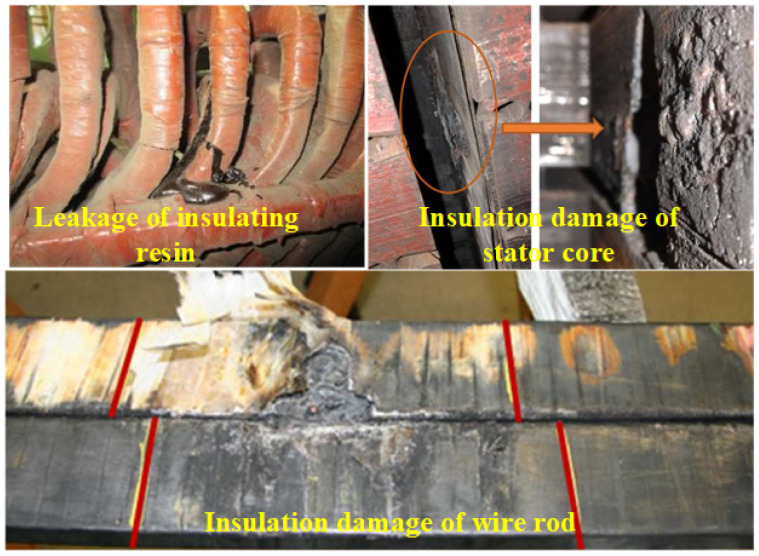
Schematic diagram of the winding fault of the hydro-generator [1].

Stator-insulation ageing begins with manufacturing flaws and is accelerated in service by thermal cycling, partial discharge, electromagnetic forces, oil, moisture, and reactive gases. These stresses enlarge micro-defects, drive resin loss, and damage both core and conductor insulation, leading to irreversible degradation that ends in turn-to-turn, phase-to-phase, or—most frequently—single-phase earth faults, as shown in Figure 1. Conventional relays cannot locate earth faults, and the strongly non-linear, saturated, and noisy environment of the hydro-generator further complicates diagnosis.

To efficiently and accurately characterize large generators under ground fault conditions, scholars have proposed various methods for modeling motor faults. In ref. [2], the circuit is divided into multiple unit structures based on the actual winding connection and multi-loop theory. A transient circuit model is then established for a single-phase ground fault. In ref. [3], a multi-loop model of the generator circuit is developed, and the fourth-order Runge–Kutta algorithm is employed to improve computational efficiency. In ref. [4], fault modeling is conducted for the 1000 MW hydro-generator used at the Baihetan Hydropower Station, analyzing fault current, transient overvoltage, and neutral point drift during a single-phase grounding fault. In ref. [5], a “double dampers per pole” principle is proposed to address the challenge of modeling numerous dampers on stator windings in large generators. This approach reduces model complexity while maintaining accuracy. In ref. [6], the propagation characteristics of traveling waves in stator windings are analyzed. A wave circuit model is established, the Karen Bell transform is applied to examine traveling wave modulus components, and wavelet transform is used to extract high-frequency information. In ref. [7], the finite element method is employed to develop an electromagnetic model of the motor and to analyze unbalanced magnetic pull caused by eccentricity and short-circuit faults. Although the finite element method offers high analytical precision and rich computational results, its application to large hydro-generators is hindered by the large volume, high hardware requirements, and slow computation speed. In ref. [8], a fault model is established using a field-circuit coupling approach: the electromagnetic field is simulated via the finite element method, while circuit methods are used to compute flux linkage and end leakage inductance matrices. These are then coupled into a parameter matrix. This method simplifies the finite element model and incorporates non-linear factors that are difficult to address with pure circuit methods. In summary, although many research results have been achieved in the study of single-phase ground faults in large generators, the existing models still exhibit limited adaptability.

After obtaining the electromagnetic characteristics of the generator following a fault by modeling the single-phase ground fault in a large generator, it is still necessary to diagnose and locate the fault in order to determine the fault type and phase. Existing methods for single-phase ground fault diagnosis and location can be categorized into algorithms based on operational data processing and algorithms based on current or voltage pulse injection. The accuracy of fault diagnosis and localization is directly linked to algorithm complexity and the sampling accuracy of operational data. Ref. [9] introduced a practical fault detection method based on the relationship between the phase of the zero-sequence voltage of the fundamental potential in the hydro-generator winding and the fault location and transition resistance. This method is simple, easy to operate, and does not require additional equipment. However, it can only accurately identify the phase of the fault location. In ref. [10], the non-linear output frequency response function is used for fault detection in hydro-generators. A fast online monitoring method is proposed and compared with the traditional least squares method, demonstrating the superiority of the proposed method in terms of diagnosis speed. The air gap flux density of the generator is monitored in ref. [11] using sensors installed in the air gap. The fault type of the generator is determined based on the air gap flux density. However, pinpointing the exact fault location is not possible. For large hydro-generators, a significant number of sensors would be needed, resulting in high costs. In ref. [12], a double hidden-layer backpropagation neural network prediction model is utilized, and a fault diagnosis algorithm with a degree of expandability is obtained through data training. In ref. [13], fuzzy theory is utilized for fault characteristic classification, while the Elman neural network is employed for fault characteristic identification and comparison with winding faults. This approach enables online monitoring of the generator; however, it is limited to fault type identification and does not determine the fault location. Ref. [14] presented a turbine fault diagnosis model using a Bayesian-optimized convolutional neural network combined with long short-term memory, which demonstrates high diagnostic accuracy. The accuracy is improved by at least 5.5% compared to previous algorithms. Ref. [15] proposes a winding fault diagnosis method for permanent magnet motors based on second-order positive/negative sequence Park transformation. It can clearly separate turn-to-turn shorts from high-resistance faults and improves both the reliability and speed of fault identification. Ref. [16] presents a real-time detection method that uses a light preprocessing network, a fine-grained feature extraction network, and a multi-task joint optimization framework. Under conditions of strong light changes, dirt, and high-speed rotation, it maintains an average angle error of 0.8° and runs at 25 FPS. This robust monitoring significantly enhances the accuracy and reliability of detecting mechanical assembly problems in generators. Ref. [17] offers a hybrid strategy that combines model-based residual generation with intelligent algorithms. By using structural analysis and optimized residual selection, it significantly improves the precision of fault detection and isolation. Ref. [18] introduces a turn-to-turn short fault index based on the amplitude and phase of positive/negative sequence currents. It requires only the three-phase currents and can detect both light and heavy faults across wide speed and load ranges without any motor parameters or voltage data. Ref. [19] puts forward a real-time method that combines structural analysis with a generalized likelihood ratio test. It can detect early turn-to-turn shorts and encoder faults in permanent magnet synchronous motors even when noise parameters are unknown. Tests show it can identify turn-to-turn shorts as low as 1%. The main issue with the methods mentioned above is that they require a strict monitoring environment, are significantly affected by noise in the sampled information, and need improvement in high-precision fault location performance.

Compared to algorithms based on operational data processing, the current or voltage injection method is a better choice for accurately locating faults. Many researchers have enhanced the current injection method to further improve the accuracy of fault positioning. Ref. [20] introduced a fault detection system that utilizes the partial discharge method to identify faults in large hydro-generators. This new system enhances anti-interference capabilities and addresses the limitations of online monitoring. However, it should be noted that the method is specifically designed for detecting short-circuit faults at the generator outlet and does not offer global fault localization in the stator. In their work, ref. [21] introduced a fault diagnosis technique based on armature reaction that is capable of adapting to the load conditions of the generator. The method relies on the high similarity of the distribution pattern of winding electromotive force under both no-load and load conditions of the motor. By adjusting the fault diagnosis evaluation function based on the fault winding voltage and measuring the phase resistance using an injection device, the method can accurately pinpoint faults under various working conditions. This approach reduces the positioning error inherent in traditional injection methods for fault monitoring. It is effective within a maximum positioning range of five turns, but it does increase the complexity of the algorithm and requires online operational data. In ref. [22], a method for fault location and fault potential calculation is presented, taking into account the association between the location of the single-phase grounding fault and the injected current. By analyzing the variation of the neutral point fundamental voltage, it becomes possible to effectively distinguish between permanent and temporary faults, thus further enhancing the precision of single-phase grounding fault location and identification. Ref. [23] presents an online turn-to-turn short detection method for interior permanent magnet motors. It injects a pulsating high-frequency voltage into the d-axis and picks out a fault-linked harmonic that is free from rotor saliency. The method reliably spots a 2% short at 300 rpm and still catches a 5% short at standstill. Ref. [24] offers an online way of finding and locating faulty nodes. It injects a high-frequency current and uses smart meters to read the high-frequency impedance at each node. Power stays on while the method pinpoints illegal loads or faults in low-voltage distribution networks. Ref. [25] builds an online diagnostic tool that monitors the zero-sequence current. An analytic model pulls out the fault signature and FFT reads the high-frequency pulses. The tool locates early, weak shorts in the rotor winding and rates their severity under changing speed and load and sub- and super-synchronous states. Ref. [26] injects two high-frequency square-wave voltages into a permanent magnet synchronous motor and compares the resulting currents. This step removes the effect of neutral-point shift and allows early, accurate grading of stator turn-to-turn shorts. Ref. [27] introduces a scheme that relies on high-frequency voltage residuals. It applies balanced three-phase high-frequency voltages and uses the standard deviation of the residuals as the fault flag. No extra hardware is needed for robust detection and location of turn-to-turn shorts. Ref. [28] works with equivalent high-frequency impedance. It takes voltage and current at the PWM switching harmonics, forms the impedance, and observes the difference between phases. Simulations on both surface-mounted and interior permanent magnet motors and tests on a triple-redundant prototype show the fault signal ratio rises about 30% above the classic high-frequency ripple method. The result is early, fast detection that is little affected by external fault resistance. Ref. [29] presents an online diagnostic method for the power converter of an SRM drive. It injects a sinusoidal high-frequency current into the upper switch of the asymmetric converter and tracks changes in frequency and amplitude. Open and short faults are caught and located right away, and no extra sensors are required to raise system reliability. The diagnostic algorithms or methods mentioned earlier are unable to hierarchically determine the fault phase, fault branch, fault coil, and fault bar of the system. The implementation process of these algorithms contains redundancy and cannot be utilized in the periodic maintenance process. Furthermore, there is considerable scope for enhancing accuracy. The advantages and disadvantages of various fault diagnosis methods are presented in Table 1.

In summary, this paper proposes a method for diagnosing and positioning single-phase grounding faults in large-generator stator windings. The method is based on graded frequency conversion pulse injection, aiming to enhance maintenance efficiency and positioning accuracy of large generators. In the second part, the structure of the large generator is given, and the finite element model is established. The operation characteristics of the large generator under normal and single-phase ground faults are analyzed. The single-phase ground fault model under low-frequency pulse and high-frequency pulse injection is established, which lays a foundation for the diagnosis and location of a single-phase ground fault. The third part gives the implementation process of the proposed single-phase grounding fault diagnosis and location method of large-generator stator winding based on stage frequency conversion pulse injection. In the fourth part, the effectiveness of the proposed method is verified by simulation, and comparative analysis is carried out. The fifth part gives the conclusion of this paper.

## 2. Modeling and Analysis of Generator Stator Single-Phase Grounding Fault

### 2.1. Generator Structure

Large hydraulic generators adopt a salient pole structure. Compared with the non-salient pole motor, the structure of the motor is more complex and the non-linear characteristics are more serious. The generator structure is a vertical-shaft semi-umbrella closed self-circulation fully air-cooled three-phase salient pole synchronous generator. The basic principle of a salient pole synchronous generator is that direct current is passed into the rotor winding to generate an exciting magnetic field, and the rotor rotates under the drive of water flow to form a rotating magnetic field. The rotating magnetic field cuts the stator windings, generates induced electromotive force, and realizes the conversion of mechanical energy to electric energy.

**Figure 2:**
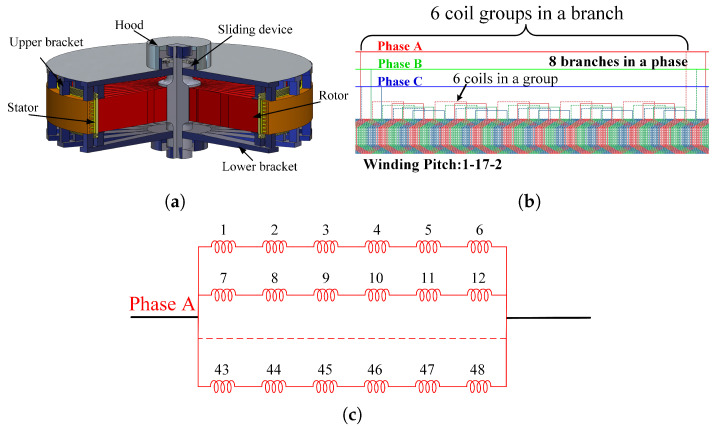
Salient pole generator structure. (**a**) Overall structure. (**b**) Partial stack winding. (**c**) Single-phase branch.

Salient pole synchronous generators adopt vertical-shaft semi-umbrella closed self-circulation air cooling; the main components include a stator, rotor, sliding device, upper guide bearing, upper bracket, lower guide and thrust bearing, lower bracket, air cooler, air brake, etc., as shown in Figure 2a. The stator core is stacked with 0.5 mm thick, high-permeability, low-loss, non-aging, high-quality cold-rolled thin silicon steel sheets, and is compressed with high-strength core bolts. Ventilation slots are arranged between the segment core, and the end of the stator core adopts a step design to reduce the magnetic field strength of magnetic leakage. The stator winding is in the form of overlapping winding, with 48 coils in each phase forming 8 branches; each branch consists of 6 coil groups, as shown in Figure 2b,c. The stator-winding bar is wound with mica tape and using the vacuum pressure impregnation method. The inner part of the groove is low-resistance and the end part is high-resistance. The rotor is mainly composed of a spindle, a rotor support, a magnetic yoke, a magnetic pole, etc. The magnetic pole is the main component of generating a magnetic field, which is mainly composed of a magnetic pole core, magnetic pole coil, damping winding and pole shoe.

### 2.2. Modeling and Analysis of Single-Phase Grounding Fault

The research shows that the stator-winding single-phase grounding fault is one of the most common fault types in salient pole generators. However, conventional single-phase grounding protection devices such as fundamental voltage protection, third-harmonic voltage saturation and low-frequency power supply injection protection do not have fault location functions and cannot determine the fault degree. However, the “dichotomy” method used in most practical projects requires manual measurement of coil insulation to determine the fault degree and fault location, which inevitably increases labor and economic costs. It is worth explaining that it is very important to model and analyze the single-phase grounding fault of a large salient pole generator to realize the accurate location of the single-phase grounding fault.

**Figure 3:**
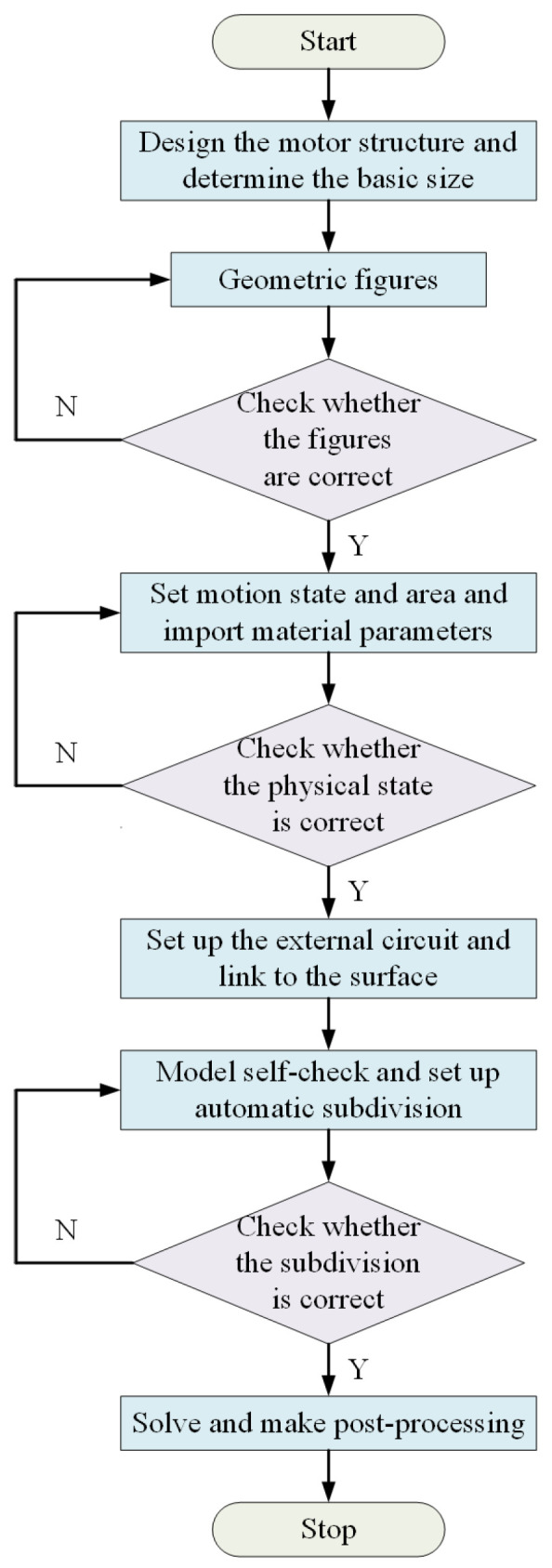
Finite element modeling process.

**Figure 4:**
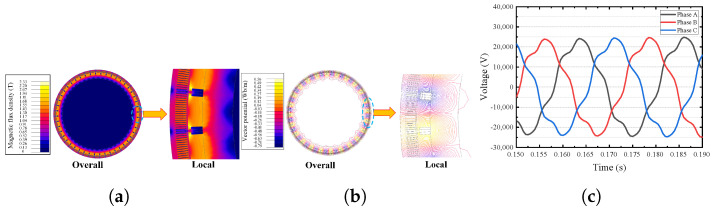
Partial electromagnetic characteristics under normal conditions. (**a**) Magnetic density distribution. (**b**) Distribution of magnetic field lines. (**c**) Induced electromotive force waveform.

**Figure 5:**
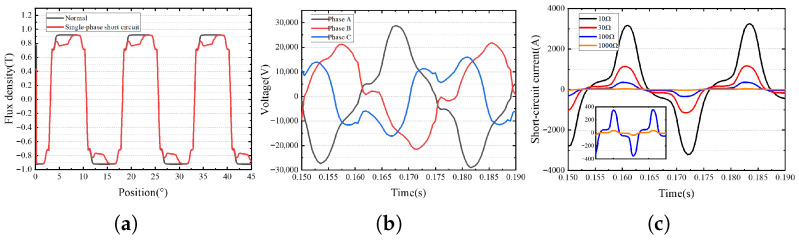
Partial electromagnetic characteristics of the single-phase ground fault case. (**a**) Magnetic density distribution under single-phase ground fault. (**b**) Electromotive force distribution under single-phase grounding fault. (**c**) Leakage current under different ground resistance.

In this paper, the finite element model of a 1000 MW stacked salient pole generator is established. The established finite element model can effectively extract the inductive electromotive force, flux linkage and inductance of the salient pole generator, which lays a foundation for the diagnosis and location modeling of single-phase grounding fault. Based on the motor design parameters given by the manufacturer, the finite element model of the motor is established according to the method shown in Figure 3. When the model is divided, automatic division and manual division are combined. Firstly, the automatic division is adopted to limit the maximum side length of the division element to no more than 1 mm. After the automatic division is completed, manual division intervention is adopted to increase the division density of the stator magnetic pole, the rotor magnetic pole and the air gap, to ensure the modeling accuracy of the finite element model. After the finite element modeling is completed, the model is post-processed and solved to obtain the distribution of magnetic density and magnetic field lines, as shown in Figure 4a,b. It can be seen that the magnetic density and magnetic force line are uniformly distributed as a whole, and the magnetic density at the stator tooth tip and the edge of the rotor slot reaches a maximum of 1.85 T, which meets the requirements of the silicon steel sheet material 50W250. Meanwhile, it can be seen from the finite element analysis results that the induced electromotive force in each phase is symmetrical, as shown in Figure 4c. After the single-phase grounding fault occurs, taking the low-resistance grounding fault of phase C at the end of coil No. 3 as an example, it can be found that the output-induced electromotive force of phase C is significantly reduced, and the three-phase asymmetric induced electromotive force is strengthened, as shown in Figure 5a,b. At the same time, if the grounding resistance is too small, there will be an obvious instantaneous leakage current, triggering protection, seriously breaking the insulation of the surrounding group and preventing safe operation, as shown in Figure 5c.

**Figure 6:**
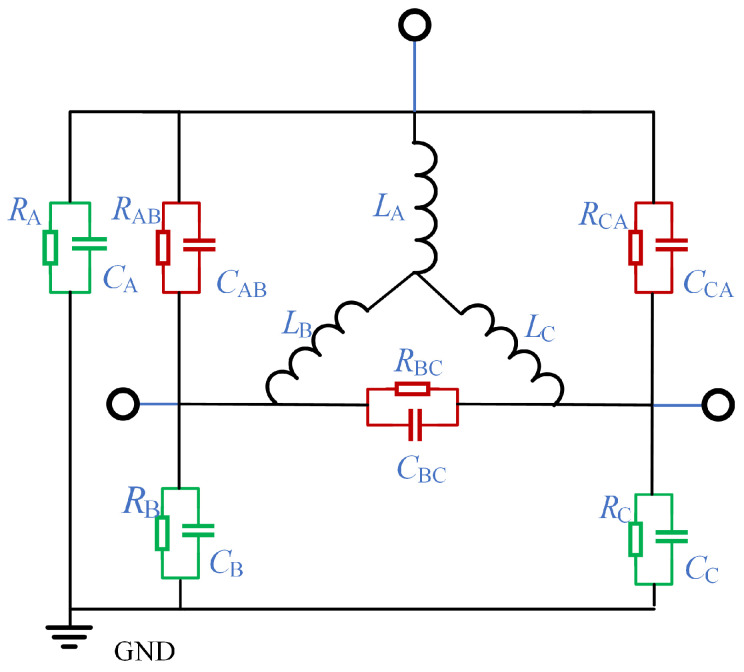
Generator insulation model.

**Figure 7:**
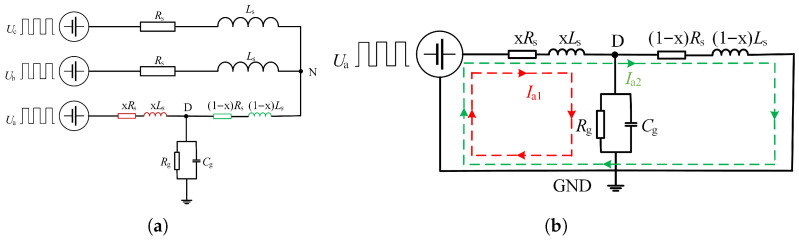
Partial electromagnetic characteristics of the single-phase ground fault case. (**a**) Single-phase grounding equivalent model at low frequency. (**b**) Single-phase grounding of two current circuits.

The insulation model of the generator is shown in Figure 6, where $L_{A}$, $L_{B}$ and $L_{C}$ are the equivalent inductance of the A-phase winding, B-phase winding and C-phase winding, respectively. $R_{A}$, $R_{B}$, $R_{C}$, $R_{\mathrm{AB}}$, $R_{\mathrm{BC}}$ and $R_{\mathrm{CA}}$ are the insulation resistance relative to A, the insulation resistance relative to B, the insulation resistance relative to C, the insulation resistance between phase A and phase B, the insulation resistance between phase B and phase C and the insulation resistance between phase C and phase A. Usually, the insulation resistance and insulation capacitance can be measured directly by the impedance tester. Considering the method based on stepwise frequency conversion pulse injection proposed in this paper, the stator single-phase ground fault equivalent model under low frequency and high frequency is needed after the stator single-phase ground fault occurs. Considering that the method proposed in this paper applies a DC voltage pulse, the influence on the ground insulation capacitance can be ignored at low frequency. Therefore, the equivalent capacitance model at low frequencies is shown in Figure 7a. $U_{a}$, $U_{b}$ and $U_{c}$ represent the DC voltage pulses applied by phase A, B and C, respectively, $R_{s}$ and $L_{s}$ represent the internal resistance and inductance of the stator respectively, and *x* represents the position where single-phase to ground short circuit occurs in phase A. When the generator rotor is stationary, and when the DC pulse is applied, the neutral point A is grounded. For healthy phase winding without single-phase grounding fault, when the voltage pulse is high-voltage, the voltage equation is shown in (1).

(1) $$U_{s}=R_{s}i_{a}+L_{s}\frac{di_{a}}{dt}$$

$i_{a}$ is the current of phase A. $U_{s}$ is the DC bus voltage, when the voltage pulse is low-voltage, the voltage equation is shown in (2).

(2) $$0=R_{s}i_{a}+L_{s}\frac{di_{a}}{dt}$$

When the stator single-phase grounding fault occurs, the current has two loops, including the main current loop and the leakage current loop. The main current loop remains unchanged, and the voltage equation of the leakage current loop is shown in (3).

(3) $$U_{s}=\left(xR_{s}+R_{g}\right)i_{g}+xL_{s}\frac{di_{g}}{dt}$$

$R_{g}$ is the ground resistance; $i_{g}$ is the leakage current. Since the insulation resistance is generally much greater than the internal resistance of the stator winding, (3) can be simplified to (4).

(4) $$U_{s}=R_{g}i_{g}+xL_{s}\frac{di_{g}}{dt}$$

Therefore, the leakage current $i_{g}$ at this time can be obtained, as shown in (5).

(5) $$i_{g}=\frac{U_{s}}{R_{g}}+\frac{xL_{s}}{R_{g}}\frac{di_{g}}{dt}$$

According to (5), $i_{g}$ is related to $U_{s}$, $R_{g}$ and $xL_{s}$. For different fault types such as metal grounding, low-resistance grounding and high-resistance grounding, the leakage current has different performance characteristics, so the leakage current can be used to evaluate the change of generator insulation resistance to the ground, and can be used as an indicator of fault characteristics.

**Figure 8:**
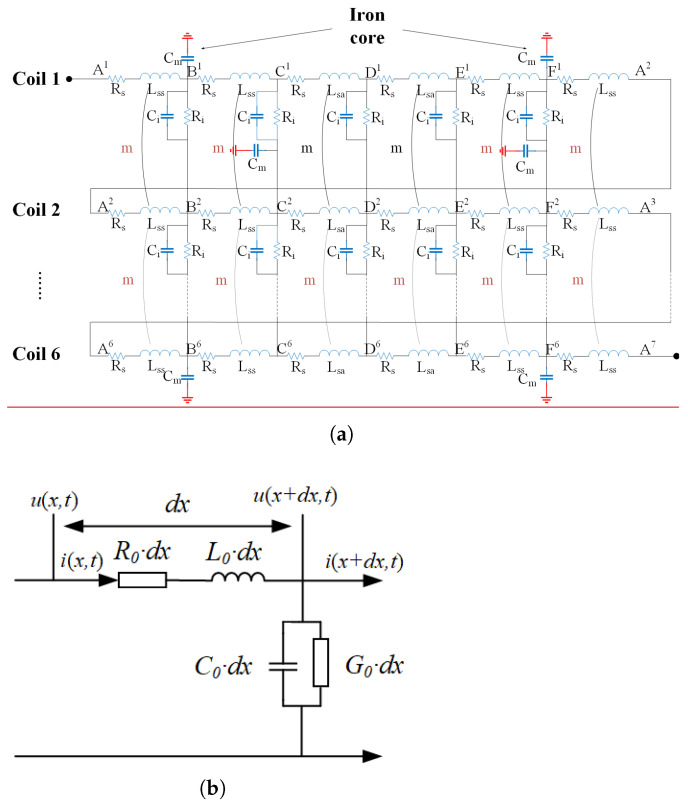
Equivalent model of single-phase grounding of generator. (**a**) Distributed parameter model at high frequency. (**b**) Equivalent uniform transmission line model.

Through the above analysis, it can be found that although the injection of a low-frequency switching signal can reflect the degree of ground short circuit of the motor by calculating *x*, it cannot accurately reflect the fault location due to the influence of stray parameters and magnetic field coupling performance, so it is necessary to establish a single-phase grounding short circuit model at high frequency. Since the concentrated parameter model used at low frequency cannot reflect the internal structure of the stator winding and does not correspond to its actual physical significance, the distributed parameter model of single-phase grounding fault under high-frequency pulse is established in this paper, as shown in Figure 8a. The modeling method is based on the uniform transmission line theory, and the equivalent uniform transmission line model of a single coil group is shown in Figure 8b. The equations shown in (6) can be obtained from Kirchhoff’s current law and Kirchhoff’s voltage law.

(6) $$\begin{array}{c} L_{0}dx\frac{\partial i(x,t)}{\partial t}+R_{0}dxi\left(x,t\right)+u\left(x+dx,t\right)=u\left(x,t\right)\\ C_{0}dx\frac{\partial u(x+dx,t)}{\partial t}+G_{0}dxu\left(x+dx,t\right)+i\left(x+dx,t\right)-i\left(x,t\right)=0 \end{array}$$

$L_{0}$ is the wave inductance, $R_{0}$ is the wave resistance, and $C_{0}$ is the wave capacitance. To solve (6), the result can be shown as (7).

(7) $$\begin{array}{c} \dot{U}\left(x\right)=A_{1}e^{-\gamma x}+A_{2}e^{\gamma x}\\ \dot{I}\left(x\right)=B_{1}e^{-\gamma x}-B_{2}e^{\gamma x} \end{array}$$

$A_{1}$, $B_{1}$ are the voltage and current amplitudes of the forward wave, $A_{2}$, $B_{2}$ are the voltage and current amplitudes of the reverse traveling wave, and $A_{1}$, $B_{1}$, $A_{2}$, $B_{2}$ are determined by the boundary conditions. When the high-frequency signal propagates on a uniform transmission line, the traveling wave will be refracted and reflected when it encounters the discontinuity of wave impedance.

## 3. The Proposed Fault Diagnosis Method

**Figure 9:**
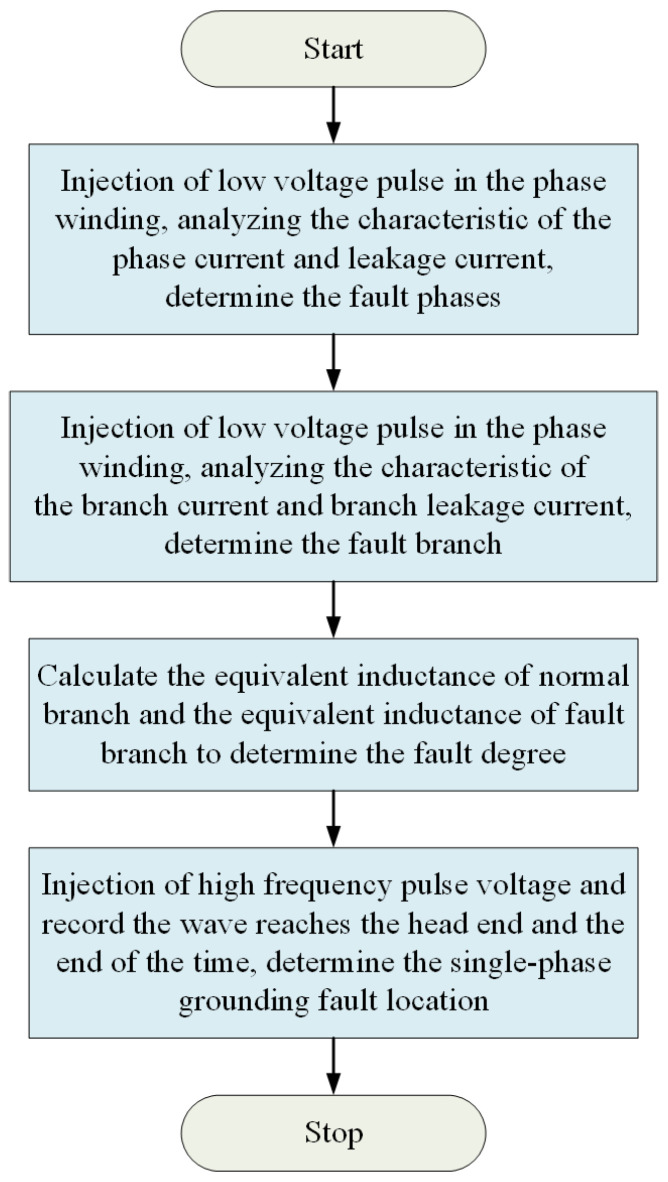
Diagnostic flow chart.

Aiming at the problems of insufficient high-frequency signal strength, inaccurate fault degree detection, and inaccurate fault location by low-frequency signals, a fault location method based on hierarchical frequency conversion pulse injection is proposed. The diagnosis process is shown in Figure 9.

**Figure 10:**
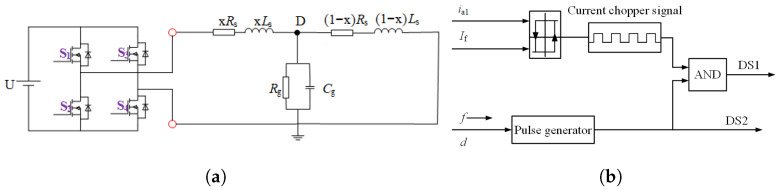
Principle of pulse generation device. (**a**) Circuit principles. (**b**) Principle of pulse generation.

Firstly, the head and end of the measured winding are connected to the pulse generator to confirm the single-phase grounding fault phase. The pulse-generating device is a single-phase full-bridge inverter circuit, as shown in Figure 10a, which can realize the positive and negative injection of pulses; that is, the injected current pulse flows from the end of the winding to the end or the end of the winding to the first end. The forward injection method is chosen in this paper. The pulse generation mode is shown in Figure 10b. The current hysteresis controller generates the driving signal of switching tube S1. In contrast, the driving signal of switching tube S2 is a periodic switching signal, which is used to control the period of injection pulse, reduce the generation loss and torque caused by current pulse injection, and prevent rotor rotation. After injecting a low-frequency voltage pulse, the first end current ($i_{h}$) and end current ($i_{e}$) of the winding are collected, and the difference is calculated to calculate the ground current ($i_{g}$), and then the characteristics of $i_{g}$ and $i_{e}$ are comprehensively analyzed to determine the fault phase and fault degree. Normally, $i_{h}$ is equal to $i_{e}$ and $i_{g}$ is equal to 0; when a single-phase grounding short circuit fault occurs, $i_{h}$ is greater than $i_{e}$, and $i_{g}$ is not equal to 0. However, after the fault, with the change in metallic ground resistance, low ground resistance and high ground resistance, a single current characteristic cannot meet the requirements of high-precision diagnosis and location. First, the characteristics of ie are analyzed. Under normal circumstances, the end currents of phase A, phase B and phase C are symmetrical. Once a single-phase grounding fault occurs, the symmetry of the current is destroyed.

**Figure 11:**
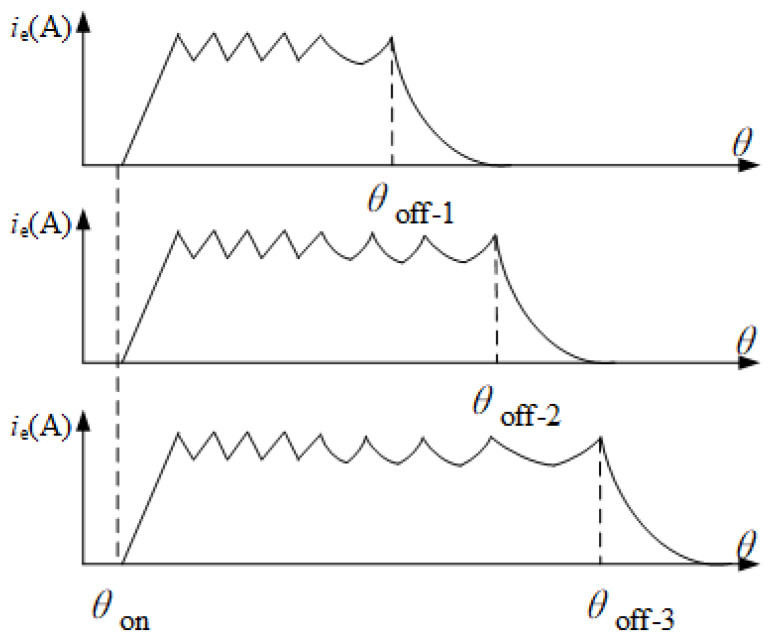
Normalization of different conduction times.

The current vector diagram is constructed with the help of the idea of rotation vectors. The core of the construction is to convert the time coordinate of the end current into polar coordinates. The current parameter changes periodically with the operation of the winding when the motor is running, and the current change in a specific period is reflected in the polar coordinate diagram to build the current vector diagram. There are overlapping regions between different phase currents, which will not only reduce the intuitiveness of the current vector diagram but also increase the difficulty of fault diagnosis. Therefore, it is considered to limit the current of each phase within 120° by using the scale reduction change. As shown in Figure 11, the corresponding angle at the beginning of the excitation of the winding is $\theta_{\mathrm{on}}$, and the corresponding angle at the moment when the winding enters the demagnetization interval is $\theta_{\mathrm{off}}$. The angle corresponding to the conduction angle $\theta_{c}$ is $\theta_{c}$ = $\theta_{\mathrm{off}}$−$\theta_{\mathrm{on}}$. The larger the conduction angle is, the larger the corresponding working interval is. To overcome the influence of different conduction intervals, a scaling variation coefficient *k* is defined in this paper, which is used to compress the operating interval of each phase to 120°. The selection of the change coefficient of scale reduction should consider the most extreme case; that is, the maximum angle of the demagnetization interval corresponding to the time when the maximum demagnetization interval occurs should be considered. The expression of the change coefficient of scale reduction *K* is shown in (8).

(8) $$K=\frac{90^{\circ }}{\theta_{c}+\theta_{\max}}$$

The maximum electrical angle of the demagnetization interval $\theta_{\max}$ can be estimated based on the current waveform. There is a statistical rule, and the selection of this value is related to CCC control, upper-limit current, winding inductance resistance and other parameters.To ensure that the working interval of each phase can be completely compressed, the value of $\theta_{\max}$ should be relatively conservative. Combined with the actual operation, the value of $\theta_{\max}$ in this study is 30°. Taking phase A as an example, the functional relation before scaling transformation is $i_{a}$ = f=(θ), and the functional relation of the current vector graph under the electrical angle after scaling transformation can be updated as follows:

(9) $$i_{a}\prime =f\left(k\theta \right),\theta \in \left(0,90^{\circ }\right]$$

**Figure 12:**
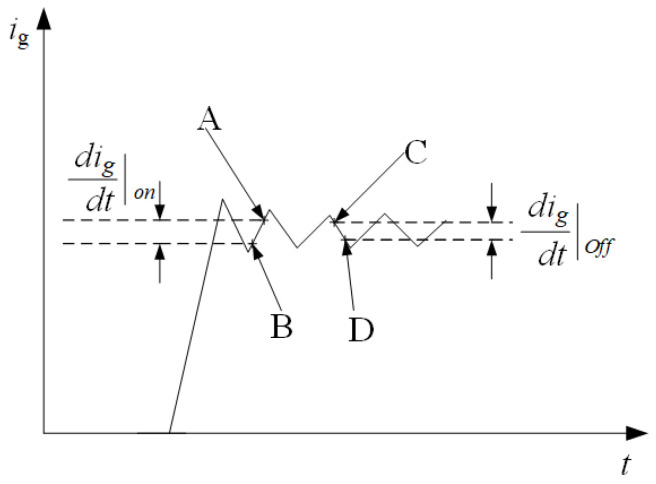
Calculation principle of leakage current.

The fault phase can be judged effectively by the above terminal current vector characteristic diagram. After the fault phase judgment is completed, enter the second part, and diagnose the single-phase grounding fault by calculating the inductance corresponding to the branch leakage current loop. The calculation principle is shown in Figure 12. At this time, the voltage equation of the leakage current loop at $t_{k-1}$ and $t_{k}$ moments is shown in Equations (10) and (11).

(10) $$\frac{di_{g}}{dt}\left|on\right.=\frac{U_{s}-\left(xR_{s}+R_{g}\right)i_{g}}{xL_{s}}$$

(11) $$\frac{di_{g}}{dt}\left|off\right.=\frac{-\left(xR_{s}+R_{g}\right)i_{g}}{xL_{s}}$$

Subtract (10) from (11) so that the result of (12) can be obtained.

(12) $$xL_{s}=\frac{U_{s}}{\frac{di_{g}}{dt}\left|on\right.-\frac{di_{g}}{dt}\left|off\right.}$$

Similarly, the inductance under normal circumstances can be calculated according to this method, and *x* can be obtained by comparing the calculation result of (12) with the value of normal inductance, to realize fault location.

The fault location can be determined by calculating the leakage inductance, and then the fault location can be further determined by the wave impedance method of high-frequency pulse injection, and the position of the branch inductance is compared, and the average value is obtained, to further improve the accuracy of fault diagnosis and location.

**Figure 13:**
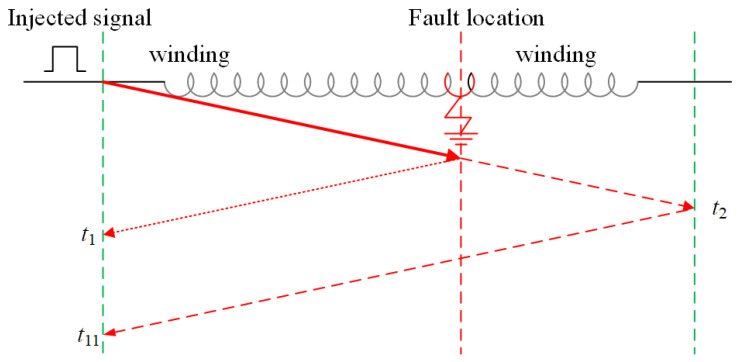
Diagnosis principle of high-frequency injection.

In the case of high-frequency pulse injection, the positioning principle is shown in Figure 13. The moment when the first high-frequency component first reflects the end is denoted as $t_{1}$, the moment when the first reaches the end is denoted as $t_{2}$, and the total length of the fault branch is denoted as *l*. The calculation formula for *x* at this time is shown in (13).

(13) $$\begin{array}{c} \left\{\begin{array}{cc} t_{1}= & \frac{2x}{v}\\ t_{2}= & \frac{l}{v}\\ x= & \frac{l}{2}\frac{t_{1}}{t_{2}} \end{array}\right. \end{array}$$

## 4. Simulation Analysis

**Figure 14:**
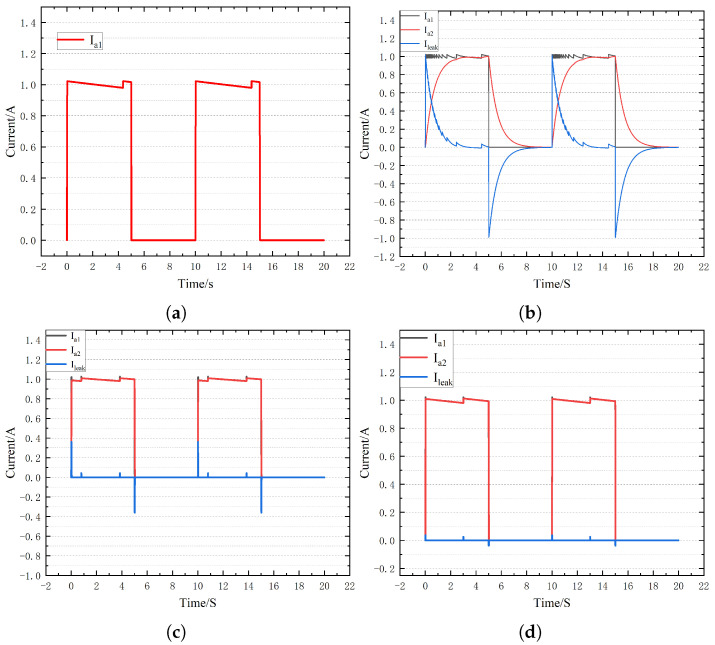
Current distribution under different ground resistances for injection current 1A and bus voltage 100 V. (**a**) Normal circumstances. (**b**) $R_{g}=1\;\Omega$. (**c**) $R_{g}=100\;\Omega$. (**d**) $R_{g}=1000\;\Omega$.

To verify the validity of the proposed fault diagnosis algorithm, simulation models of large salient pole generators under normal and ground faults are established in Matlab/Simulink. We set the injection voltage amplitude to 100 V, the current limit to 1 A, the current ring width to 0.2 A, the pulse generator frequency to 0.1 Hz, and the duty cycle to 0.5. Figure 10 shows the changes in the head current, end current and leakage current under normal circumstances with $R_{g}$ equal to 1 Ω, 100 Ω and 1000 Ω. As can be seen from Figure 14a, under normal circumstances, the head current and the end current are equal, and the leakage current is equal to 0. When Rg is equal to 1 Ω, there is a significant difference between the end current and the head current, and it decreases gradually in the form of the chopper, which lays a foundation for the calculation of leakage current loop inductance. At the same time, with the increase in $R_{g}$, the leakage current gradually decreases, and the end current and the head current tend to be the same, but the characteristics of the leakage current are different from the normal circumstances.

**Figure 15:**
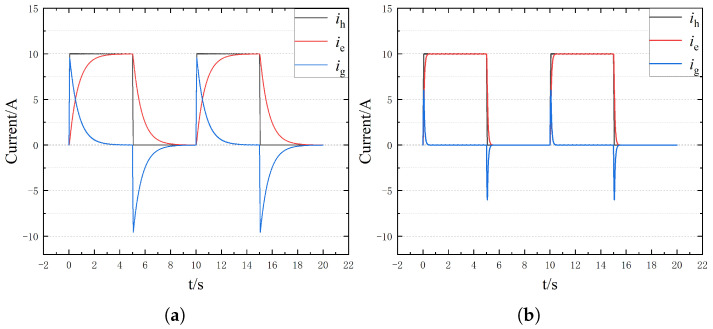

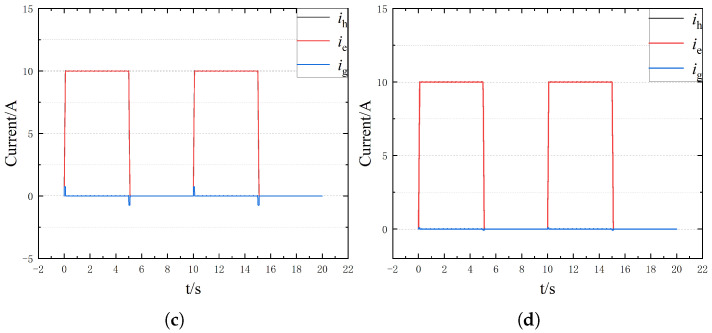
Current distribution under different ground resistances when the injection current is 10 A and the bus voltage is 200V. (**a**) $R_{g}=1\;\Omega$. (**b**) $R_{g}=10\;\Omega$. (**c**) $R_{g}=100\;\Omega$. (**d**) $R_{g}=1000\;\Omega$.

To further the ability of the proposed method to extract fault characteristics, the bus voltage is set to 200 V and the injection current to 10 A. The changes in current characteristics under different ground AH resistances are shown in Figure 15. As can be seen from the figure, with the increase in bus voltage and injection current amplitude, the characteristics of end current and leakage current become more obvious.

**Figure 16:**
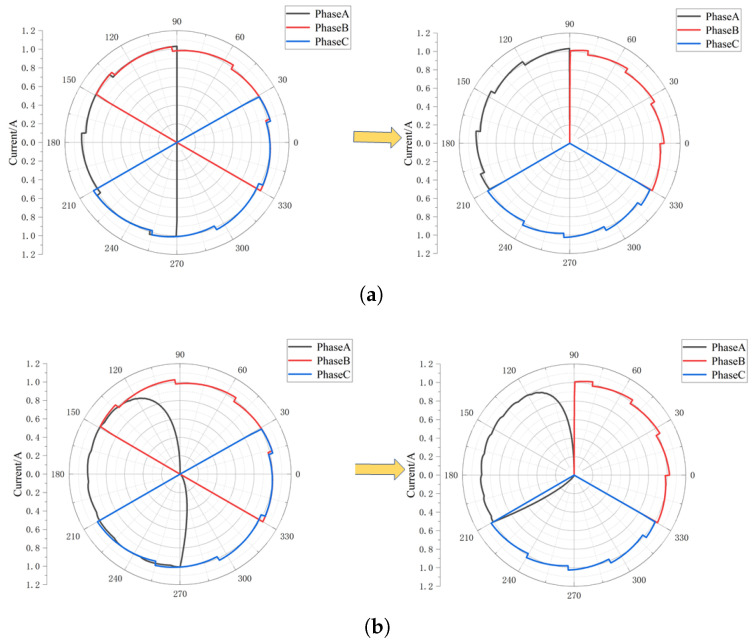

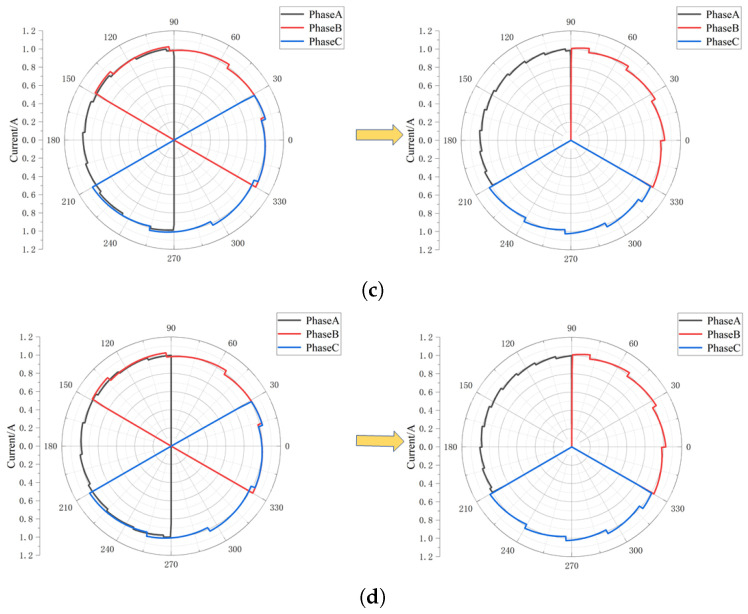
Terminal current vector diagram with injection current 1 A and bus voltage 100 V. (**a**) Normal circumstances. (**b**) $R_{g}=1\;\Omega$. (**c**) $R_{g}=100\;\Omega$. (**d**) $R_{g}=1000\;\Omega$.

**Figure 17:**
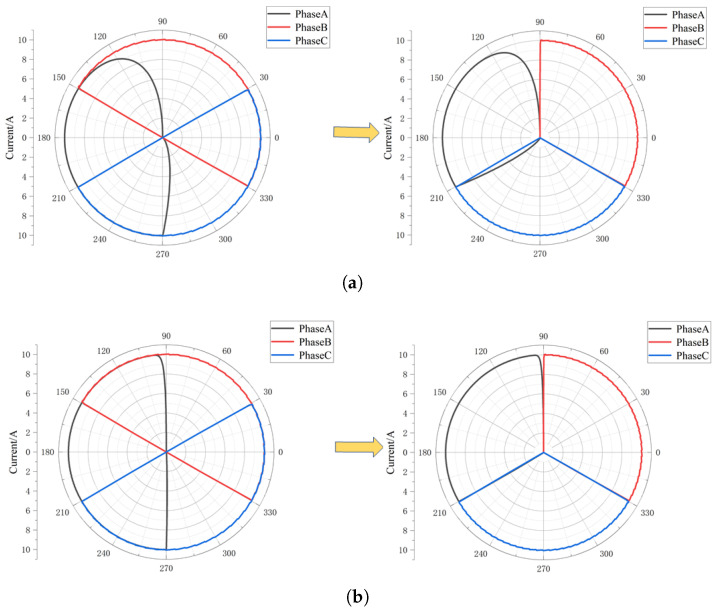

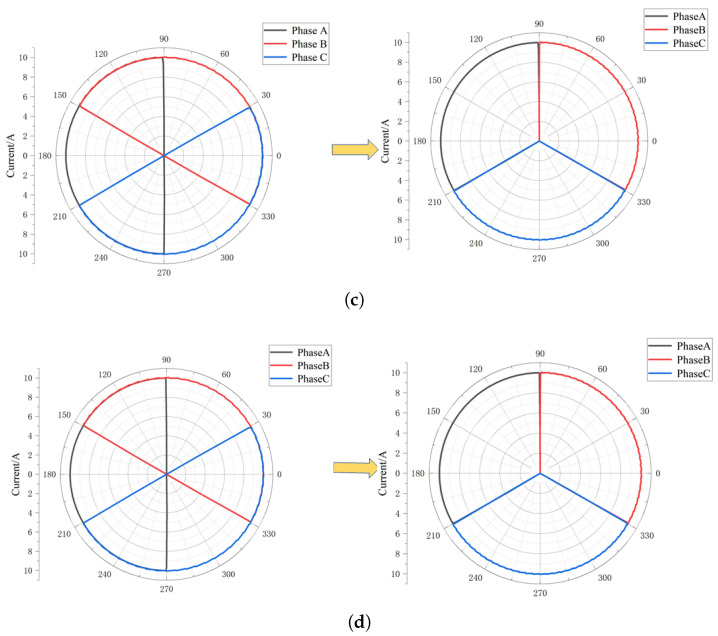
Terminal current vector when injection current is 10 A and bus voltage is 200 V. (**a**) $R_{g}=1\;\Omega$. (**b**) $R_{g}=10\;\Omega$. (**c**) $R_{g}=100\;\Omega$. (**d**) $R_{g}=1000\;\Omega$.

Based on the data in Figure 14 and Figure 15, the fault diagnosis and location method proposed in this paper based on the terminal current vector diagram is adopted, and the terminal current vector diagram is obtained as shown in Figure 16. As can be seen from Figure 16a, under normal circumstances, the terminal current symmetry is good. At the same time, under the action of the current interval compression algorithm, the end current of each phase is compressed to 120° electrical angle, and the current characteristics of each phase vector have no influence and are more visible. With the occurrence of a single-phase short-circuit fault to the ground, the symmetry of the end current is destroyed; especially when the resistance to the ground is small, the asymmetry of the three-phase current is more obvious. At the same time, with the increase in injection current and bus voltage, the characteristics of end vector current become more clear, especially when the ground resistance is large, as shown in Figure 17. For example, by comparing Figure 16d and Figure 17d, it can be seen that the asymmetry of the end current vector at this time is significantly reduced in Figure 16d, while it is significantly strengthened in Figure 17d, which is consistent with the above analysis. Therefore, the fault phase of single-phase grounding can be obtained.

**Figure 18:**
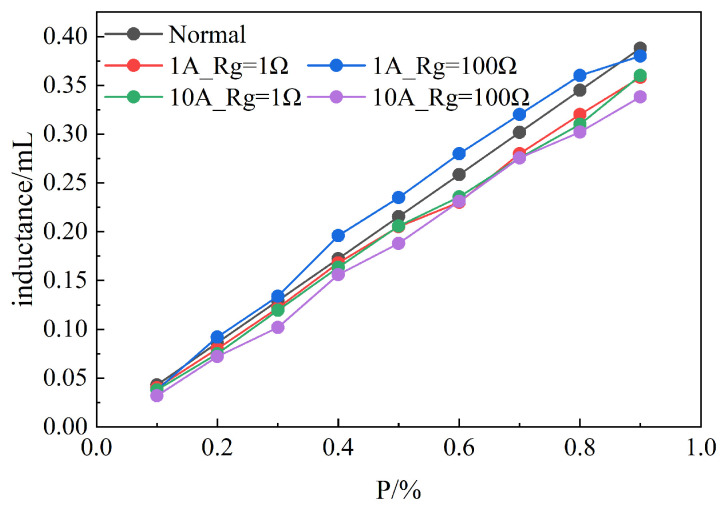
Inductance distribution of leakage current loop at different fault locations.

After determining the fault phase, a low-frequency current pulse is injected by the same method, and the equivalent inductance of the leakage current loop under different branches of the fault phase is calculated by combining Formula (11) and Formula (12), as shown in Figure 18. It can be seen from Figure 18 that the proposed method can calculate the corresponding leakage current loop inductance following the change of fault location under different $R_{g}$ and injection current levels. At the same time, it can be seen that the calculation accuracy of the leakage loop inductance is higher when the grounding resistance is small, such as the metallic grounding resistance. With the increase in the grounding resistance, the calculation accuracy of the leakage loop inductance decreases, but the overall trend remains unchanged. With the increase in injection current amplitude, the calculation precision of leakage current loop inductance increases, which is consistent with the theoretical analysis above. Therefore, the proposed method based on equivalent inductance calculation of leakage current loop can effectively determine the single-phase grounding fault branch.

**Figure 19:**
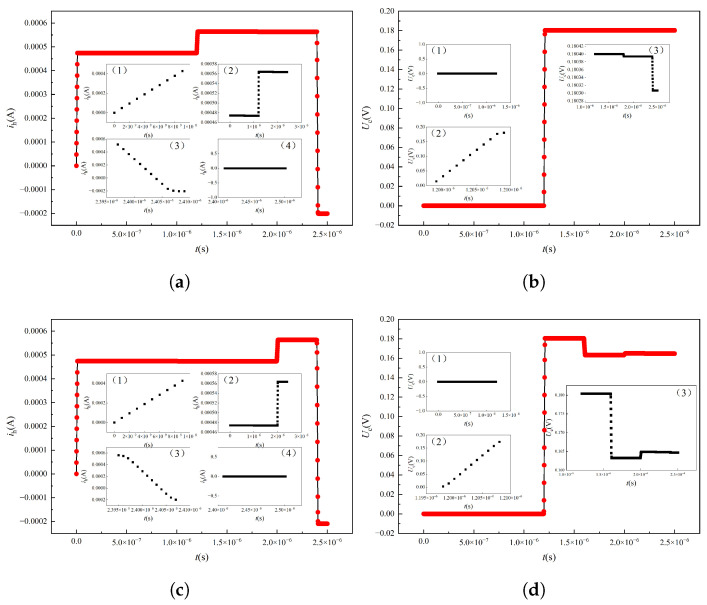
Signals at the lower head and end of the HF injection for different fault locations. (**a**) The first end current at 180 m. (**b**) Terminal voltage signal at 180 m. (**c**) The first end current signal at 300 m. (**d**) Terminal voltage signal at 300 m.

After the fault branch is determined, the high-frequency fault model is established according to the method described above, in which the bus voltage is consistent with the low-frequency method. In the case of overlapping windings, the total length of a single line rod is set to 360 m, and high-frequency pulses are injected at the head end. When the single-phase grounding fault point is 180 m, the first end current signal and the end voltage signal are shown in Figure 19a and Figure 19b respectively. As can be seen from the figure, both the first end current signal and the end voltage signal have a certain degree of sudden change due to the occurrence of ground fault. At the same time, when the single-phase grounding fault point is 300 m, the current signal at the first end and the voltage signal at the end also show a certain degree of mutation, and the mutation position changes relative to that at 180 m, as shown in Figure 19c and Figure 19d respectively. Therefore, the fault location can be realized by extracting the signal mutation point under the high-frequency signal injection.

**Figure 20:**
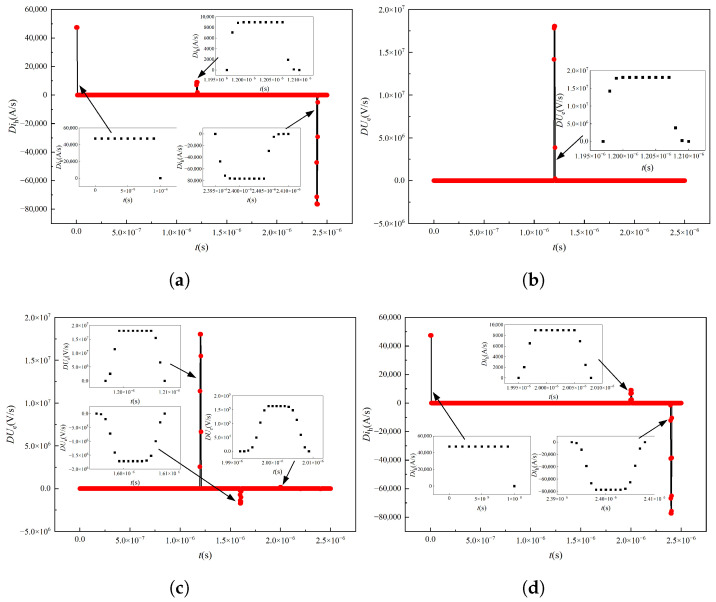
Solution of the signal slopes at the head and end under high-frequency injection. (**a**) First end current slope at fault position 180 m. (**b**) Terminal voltage slope at fault position 180 m. (**c**) First end current slope at 300 m fault position. (**d**) Terminal voltage slope at fault position 300 m.

To extract abrupt fault points, speed up the diagnosis and location of single-phase grounding faults, and avoid the use of complex algorithms such as wavelet transform, neural network and machine learning, a fault diagnosis method based on current and voltage signal slope is proposed in this paper. The calculation results of the current slope are shown in Figure 16. Based on Figure 20a, excluding the initial and terminal mutations of the first-end current signal, it can be concluded that the first-end current signal mutated at 1.171 microseconds. At the same time, it can be seen from Figure 20b that the voltage signal mutates at 1.178 microseconds. Then, based on Formula (13), it can be seen that the single-phase grounding fault occurs at 178.78m. Compared with the fault location set at 180 m, the error is 1.22 m, which is less than 1%. As can be seen from Figure 20c,d, the abrupt change time of the first end current signal and the end voltage signal is 1.99 microseconds and 1.18 microseconds respectively, and the calculated single-phase ground fault occurs at 300.80 m, which is 0.8 m error compared with the set fault position, also less than 1%. To maximize the effectiveness of the proposed method, additional fault points are set, as shown in Table 2. It can be obtained that the error of the fault occurrence point and the anchoring point at this time is less than 1%, so the effectiveness of the proposed method can be proved.

## 5. Conclusions

In this paper, the finite element model of a large hydro-generator is established, the distribution of magnetic density and electromotive force under normal and single-phase grounding faults is analyzed, and the characteristics of single-phase grounding faults are summarized. Then the equivalent circuit model of single-phase grounding fault suitable for low-frequency pulse and high-frequency pulse injection is established. On this basis, it is proposed to use the low-frequency terminal current vector feature to judge the fault phase, the low-frequency leakage current loop equivalent inductance feature to diagnose the fault branch and the high-frequency voltage-current signal slope to locate the fault point, and then realize the effective diagnosis and location of the fault phase, fault branch and fault rod under the stepwise frequency conversion pulse injection. The simulation results demonstrate exceptional fault location accuracy of the proposed method. As shown in Table 2, the positioning errors for various fault locations are consistently below 1.5 m, with percentage errors remaining under 1% across all test cases. Specifically, for fault locations at 60 m, 120 m, 180 m, 240 m, and 300 m, the method achieved errors of −0.59 m (0.98%), −0.8 m (0.66%), −1.22 m (0.67%), −1.4 m (0.58%), and 0.8 m (0.26%) respectively. This high-precision performance, with all percentage errors below 1%, validates the effectiveness and reliability of the proposed single-phase grounding fault diagnosis and location approach, confirming its practical applicability in real-world power system protection scenarios. Due to space limitations, we will, in future work, further investigate how to reduce errors that may arise from sensors, data acquisition, and data processing in practical applications. Furthermore, this paper focuses on locating faults in large hydroelectric generators that have already shut down due to failure. In future work, we will study generators that are still running, aiming to reduce the failure rate. Additionally, we plan to extend our research to multi-point fault detection, which will enhance the diagnostic capability for complex fault scenarios and improve the overall reliability of hydroelectric generating units.

## Figures and Tables

**Table 1:** Comparison of different fault detection methods.

	Third-Harmonic	Low-Freq.
**Technical Features**	**Voltage Method**	**Injection**
**Fault Location**	Detects only, no location	Limited, phase only
**Accuracy and Error**	Low, harmonic saturation	Moderate, ground resistance
**Noise Immunity**	Poor in non-linear env.	Moderate, parameter interference
**Computational Complexity**	Low, real-time harmonic	Moderate, iterative
**Hardware Requirements**	Low, integrated	Moderate, injection equipment
	**Impedance**	**Step Freq.**
**Technical Features**	**Method**	**Pulse Injection**
**Fault Location**	Node location, needs network	Hierarchical: Phase→Branch→Point
**Accuracy and Error**	Variable, impedance-dependent	High, error <1%
**Noise Immunity**	Moderate, HF noise-sensitive	Excellent, stepwise reduces noise
**Computational Complexity**	High, needs high sampling	Low, hierarchical simplification
**Hardware Requirements**	High, extra sensors	Low, simple full-bridge

**Table 2:** Comparative analysis.

Fault Location/m	Location of Location/m	Error/m	Percentage Error/%
60	59.41	−0.59	0.98%
120	119.2	−0.8	0.66%
180	178.78	−1.22	0.67%
240	238.6	−1.4	0.58%
300	300.8	0.8	0.26%

## Data Availability

The original contributions presented in this study are included in the article. Further inquiries can be directed to the corresponding author.

## Abbreviations

The following abbreviations are used in this manuscript:

$L_{A}$	Equivalent inductance of the A-phase winding
$L_{B}$	Equivalent inductance of the B-phase winding
$L_{C}$	Equivalent inductance of the C-phase winding
$R_{A}$	Insulation resistance relative to A-phase winding
$R_{B}$	Insulation resistance relative to B-phase winding
$R_{C}$	Insulation resistance relative to C-phase winding
$R_{AB}$	Insulation resistance between phase A and phase B
$R_{BC}$	Insulation resistance between phase B and phase C
$R_{CA}$	Insulation resistance between phase C and phase A
$U_{a}$	DC voltage pulse applied to phase A
$U_{b}$	DC voltage pulse applied to phase B
$U_{c}$	DC voltage pulse applied to phase C
$R_{s}$	Internal resistance of the stator
$L_{s}$	Internal inductance of the stator
*x*	Position where a single-phase to ground short circuit occurs in phase A
$U_{s}$	DC bus voltage
$i_{a}$	Current of phase A
$R_{g}$	Ground resistance
$C_{g}$	Ground capacitance
$i_{g}$	Ground current
$R_{0}$	Surge impedance (wave resistance)
$L_{0}$	Surge inductance (wave inductance)
$C_{0}$	Surge capacitance (wave capacitance)
$A_{1}$	Voltage amplitude of the forward traveling wave
$B_{1}$	Current amplitude of the forward traveling wave
$A_{2}$	Voltage amplitude of the reverse traveling wave
$B_{2}$	Current amplitude of the reverse traveling wave
*K*	Scale reduction change coefficient
$\theta_{c}$	Angle corresponding to the conduction angle
$\theta_{on}$	Angle corresponding to the start of winding excitation
$\theta_{off}$	Angle when the winding enters the demagnetization interval
$\theta_{max}$	Maximum electrical angle of the demagnetization interval
$i_{h}$	Current at the first end (head current)
$i_{e}$	Current at the end (end current)
